# Diagnostic Accuracy of Endoscopic Ultrasonography Versus the Gold Standard Endoscopic Retrograde Cholangiopancreatography in Detecting Common Bile Duct Stones

**DOI:** 10.7759/cureus.12162

**Published:** 2020-12-19

**Authors:** Mohsin Anwer, Muhammad Sohaib Asghar, Sheeraz Rahman, Shanil Kadir, Farah Yasmin, Dania Mohsin, Rumael Jawed, Gul Muhammad Memon, Uzma Rasheed, Maira Hassan

**Affiliations:** 1 General Surgery, Liaquat National Hospital, Karachi, PAK; 2 Internal Medicine, Dow University of Health Sciences, Karachi, PAK; 3 Gastroenterology, Liaquat National Hospital, Karachi, PAK; 4 Internal Medicine, Liaquat National Hospital, Karachi, PAK

**Keywords:** endoscopic ultrasound (eus), endoscopic retrograde cholangiopancreatography (ercp), gastroentero-hepatology, cbd stone, dilated common bile duct, jaundice cholestatic, sensitivity, specificity, receiver operating analysis, biliary obstruction

## Abstract

Background and objectives

Stone in the biliary tract is one of the most common causes of hospitalization. However, it is difficult to determine the prevalence of gallstones in the general population because they are often asymptomatic. Thus, management lies in the proper clearance of the common bile duct (CBD) along with the removal of the gallbladder, for which it must be diagnosed on time with proper accuracy. Imaging modalities including magnetic resonance cholangiopancreatography (MRCP), endoscopic ultrasound (EUS), and endoscopic retrograde cholangiopancreatography (ERCP) provide true visualization of choledocholithiasis with comparable sensitivities. The gold standard ERCP is an invasive procedure and may cause complications, such as pancreatitis, perforation, and bleeding. EUS is a minimally invasive procedure to assess the biliary tract using high-frequency sound waves. Until now the EUS has not been addressed much in our local tertiary care setups and this study was conducted to evaluate its accuracy in the diagnosis of choledocholithiasis. The objective of our study is to determine the diagnostic accuracy (specificity and sensitivity) of EUS versus ERCP for the diagnosis of choledocholithiasis.

Materials and methods

This retrospective study was conducted on patients suspected of having choledocholithiasis undergoing both EUS and ERCP based on their history, clinical symptoms, and laboratory test results including upper abdominal pain, deranged liver function enzymes, and a dilated CBD on radiology. EUS was initially performed for the diagnosis of extrahepatic biliary obstruction followed by one or more of the confirmatory criterion standard tests (including ERCP). In order to reduce the chances of passage of stone resulting in negative analysis, only those patients were included in which both procedures were conducted temporally close together (24-72 hours in most instances). The main outcome measures were diagnostic accuracy with the help of sensitivity, specificity, positive predictive value (PPV), and negative predictive value (NPV) using a receiver operating characteristic curve. A total of 123 patients met the inclusion criteria via non-probability consecutive sampling methods.

Results

The mean age of our study population was 50.30 ± 13.91. We included 63 males (51.2%) and 60 females (48.8%). The most frequent indication for undergoing diagnostic procedures was deranged liver function tests (67.47%). The frequent comorbidities reported were hypertension (29.26%), diabetes (21.95%), chronic liver disease (16.26%), and ischemic heart disease (4.87%). Mean alkaline phosphatase and gamma-glutamyl transferase levels were markedly raised from the baseline in the study population. Post-ERCP complications were also reported in some of the study participants. About 85 patients (69.10%) were diagnosed with choledocholithiasis among the study participants. The diagnostic accuracy of EUS was compared with ERCP revealed an area under the curve (AUC) of 0.930, standard error of 0.031, 95% confidence interval of 0.868-0.991, the sensitivity of 89.5%, specificity of 96.5%, positive predictive value of 91.9%, and negative predictive value of 95.3%.

Conclusion

It is recommended that ERCP can be selectively conducted or excluded in patients with biliary obstruction in case of EUS negative, thus minimizing the complications and morbidity associated with an invasive procedure, with our results showing a comparative diagnostic accuracy of EUS.

## Introduction

Stone obstructing the biliary tract is one of the most common diseases resulting in hospitalization. However, it is difficult to determine the prevalence of gallstones in the general population because they are often asymptomatic. Only one-third of gallstones cause symptoms or lead to complications, such as choledocholithiasis (15-20%) [1]. Thus, management lies in the proper clearance of the common bile duct (CBD) along with the removal of the gallbladder, for which it must be diagnosed on time with proper accuracy. The sensitivity and specificity of imaging techniques are critical for the diagnosis of choledocholithiasis. Imaging techniques should be able to track the presence of small stones in the bile duct. The sensitivity and specificity of imaging techniques are critical for the diagnosis of choledocholithiasis. Imaging techniques should be able to track the presence of small stones in the bile duct. Despite the high sensitivity of transabdominal ultrasound (TUS) for diagnosing cholelithiasis, identifying choledocholithiasis with TUS is difficult at times [1]. Computerized tomography scan has a greater sensitivity than TUS for diagnosing choledocholithiasis, but the level of radiation and cost has limited its use as the first-line tool for diagnosing choledocholithiasis. Non-surgical imaging modalities including magnetic resonance cholangiopancreatography (MRCP), endoscopic ultrasound (EUS), and endoscopic retrograde cholangiopancreatography (ERCP) provide true visualization of choledocholithiasis with comparable sensitivities [2].

The gold standard test is endoscopic retrograde cholangiopancreatography which has the advantage of permitting intervention if a common bile duct stone is present. However, ERCP is invasive and may miss small stones and may cause complications such as pancreatitis, perforation, and bleeding [3]. Thus, it is frequently desirable to confirm the presence of choledocholithiasis before embarking upon intervention for CBD stone removal. Endosonography combines two modalities, namely endoscopic visualization, and high-frequency ultrasound. It is commonly used in the west and we are not much familiar with this modality. Small stones, bile sludge, and even microlithiasis may be detected as compared to other non-invasive procedures. This along with safety and absent radiation makes it a reliable and excellent method for examining the biliary tract [4].

EUS is a minimally invasive procedure to assess digestive (gastrointestinal) and lung diseases. A special endoscope uses high-frequency sound waves to produce detailed images of the lining and walls of the digestive tract, chest, and nearby organs such as the pancreas, liver, and lymph nodes [5]. ERCP is a procedure that directly allows visualization of pancreatic and bile ducts. Until now the EUS has not been addressed much in our local tertiary care setups and this study was done to evaluate its accuracy in the diagnosis of choledocholithiasis. There are certain limitations of EUS like high cost, lack of trained personnel, and limited setups offering this service. Therefore, the objective of our study is to determine the diagnostic accuracy (specificity and sensitivity) of EUS versus ERCP (gold standard) for the diagnosis of choledocholithiasis.

## Materials and methods

This retrospective study took place in the general surgery and gastroenterology departments of Liaquat National Hospital, a tertiary care hospital having 700 beds with 32 specialty services, located in the heart of city Karachi, Pakistan. Data collection was commenced after approval from the hospital's research and ethics committee. A pool of patients was searched through the hospital's health management information system (HMIS) suspected of having choledocholithiasis undergoing both EUS and ERCP based on their history, clinical symptoms, and laboratory test results including upper abdominal pain, deranged liver function enzymes, and a dilated CBD on plain abdominal ultrasound or other radiological modalities. Collection of data was subsequently done involving choledocholithiasis patients who underwent EUS initially and later, ERCP was performed. The diagnostic accuracy of EUS was determined by comparing it to ERCP (taken as a gold standard) [5]. Data collection included radiological investigations (ultrasound, MRCP, CT scan, EUS, ERCP) and laboratory parameters (descriptive components of liver function tests). In order to reduce the chances of passage of stone resulting in negative analysis, only those patients were included in which both procedures were conducted temporally close together (24-72 hours in most instances). The main outcome measures were diagnostic accuracy with the help of sensitivity, specificity, PPV, and NPV. The sample size was calculated by taking a sensitivity of 96%, specificity of 57%, prevalence of 18.4%, d=10%, and a confidence level of 95%. The calculated sample size was 117 patients (Dr. Lin Naing's sample size calculator was used) [6].

The inclusion criteria were all the adult patients undergoing both EUS and ERCP for suspected choledocholithiasis. The exclusion criteria were patients undergoing either EUS or ERCP (only one of them), or patients having malignancy and undergoing ERCP for diagnostic purposes, and patients with a contraindication for ERCP. All those patients who met the inclusion criteria were included in this study via non-probability consecutive sampling methods. All the data was retrieved through the electronic medical record (EMR) system and through the HMIS department and subsequently assembled and analyzed over the software IBM Statistical Package for the Social Sciences (SPSS) for Windows, version 25.0 (IBM Corp., Armonk, NY). After descriptive statistics, the receiver operating characteristic curve was generated to determine the sensitivity, specificity, PPV, and NPV of the outcome variable.

## Results

A total of 123 patients met the inclusion criteria. The mean age of our study population was 50.30 ± 13.91. This study included 63 males (51.2%) and 60 females (48.8%). The most common indication for undergoing diagnostic procedures was deranged liver function tests (67.47%). Frequent comorbidities reported were hypertension (29.26%), diabetes (21.95%), chronic liver disease (16.26%), and ischemic heart disease (4.87%). Mean bilirubin, alkaline phosphatase, and gamma-glutamyl transferase levels were significantly raised from the normal range in the study population. Post-ERCP complications reported by the study population are mentioned in Table 1.

**Table 1:** Showing baseline characteristics of studied patients (n=123). Cutt-off ranges (above-normal levels): Total bilirubin (mg/dL) - >1.0 mg/dL Direct bilirubin (mg/dL) - >0.3 mg/dL Aspartate aminotransferase (IU/L) - >45 IU/L Alanine aminotransferase (IU/L) - >45 IU/L Gamma-glutamyl transferase (IU/L) - >55 IU/L Alkaline phosphatase (IU/L) - >135 IU/L ERCP: endoscopic retrograde cholangiopancreatography; SD: standard deviation

Variables	Mean ± SD/Frequency (percentage)
Mean age	50.30 ± 13.91
Gender	Males: 63 (51.2%)
	Females: 60 (48.8%)
Deranged liver function tests	Yes: 83 (67.47%)
	No: 40 (32.52%)
Comorbidities	Hypertension: 36 (29.26%)
	Diabetes: 27 (21.95%)
	Chronic liver disease: 20 (16.26%)
	Ischemic heart disease: 6 (4.87%)
	Asthma/Chronic pulmonary disease: 4 (3.25%)
	Chronic kidney disease: 3 (2.43%)
	Autoimmune disease: 1 (0.81%)
Laboratory values	
Total bilirubin (mg/dL)	2.83 ± 1.65
Direct bilirubin (mg/dL)	1.91 ± 0.84
Aspartate aminotransferase (IU/L)	64.59 ± 25.87
Alanine aminotransferase (IU/L)	53.47 ± 21.76
Gamma-glutamyl transferase (IU/L)	132.85 ± 86.02
Alkaline phosphatase (IU/L)	321.54 ± 187.91
Post ERCP complications	
Upper Abdominal pain	25 (20.32%)
Acute pancreatitis (with elevated amylase/lipase)	5 (4.06%)
Elevated liver enzymes (new-onset)	3 (2.43%)
Cholangitis	2 (1.62%)
Intestinal perforation	1 (0.81%)
Post-sphincterotomy bleeding	1 (0.81%)
Septicemia	1 (0.81%)
Death	0 (0.0%)

**Figure 1:**
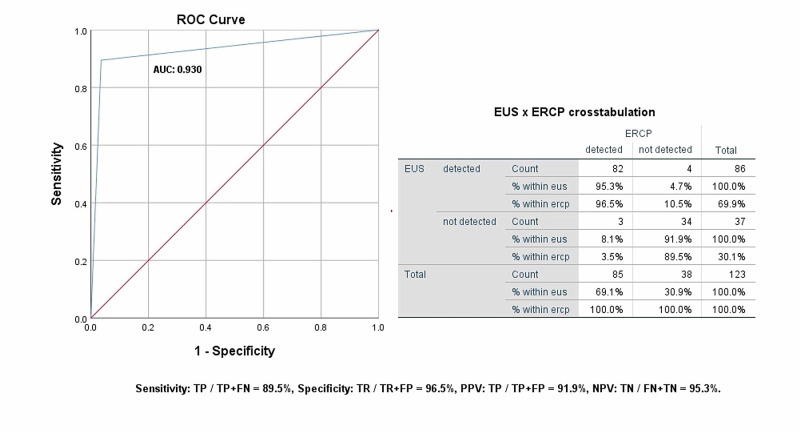
ROC curve showing diagnostic accuracy of EUS versus gold standard ERCP. EUS: endoscopic ultrasound; ERCP: endoscopic retrograde cholangiopancreatography; ROC: receiver operating characteristic; AUC: area under the curve; PPV: positive predictive value; NPV: negative predictive value; TP: true positive; TN: true negative; FP: false positive; FN: false negative

About 85 patients (69.10%) were diagnosed with choledocholithiasis among the study participants. The diagnostic accuracy of EUS was compared with ERCP using the Receiver operating characteristics analysis which revealed an area under the curve (AUC) of 0.930, standard error of 0.031, 95% confidence interval of 0.868-0.991, sensitivity of 89.5%, specificity of 96.5%, PPV of 91.9%, and NPV of 95.3%, as shown in Figure 1.

## Discussion

The various literature concluded the diagnostic accuracy of EUS versus other modalities in detecting biliary obstructive disease, as mentioned in Table 2.

**Table 2:** Showing the diagnostic accuracy of endoscopic ultrasound fetched from previously studied articles. ERCP: endoscopic retrograde cholangiopancreatography; MRCP: magnetic resonance cholangiopancreatography; IOC: intraoperative cholangiogram; TUS: transabdominal ultrasound

Author	Year of Publication	Number of study participants	Opposite diagnostic modality	Sensitivity	Specificity	Positive predictive value	Negative predictive value
Makmun et al. [6]	2017	62	MRCP, ERCP	96%	57%	88%	80%
Prachayakul et al. [7]	2014	93	ERCP	100%	80%	-	-
Tse et al. [8]	2008	2673	ERCP, IOC	94%	95%	-	-
Karakan et al. [9]	2008	120	ERCP	91%	100%	-	-
Bansal et al. [10]	2017	170	ERCP	100%	-	-	-
Netinatsunton et al. [11]	2016	141	ERCP	97.64%	80%	97.64%	80%
Wang et al. [12]	2016	202	ERCP	100%	92.88%	98.21%	100%
Ney et al. [13]	2005	215	ERCP	92-96%	100%	-	-
Lin and Huang [14]	2012	30	ERCP	100%	94.7%	91.7%	100%
Garrow et al. [15]	2007	3532	ERCP, MRCP, IOC	88-89%	90-94%	-	-
Chak et al. [16]	1999	36	ERCP, TUS	91%	100%	100%	95%
Liu et al. [17]	2001	100	ERCP, TUS	97%	98%	-	-
Polkowski et al. [18]	1999	52	ERCP, CT cholangiography	91%	100%	-	-
de Ledinghen et al. [19]	1999	43	MRCP, ERCP	100%	95%	90%	100%
Palazzo et al. [20]	1995	422	ERCP	94.9%	97.8%	100%	97%
Aubertin et al. [21]	1996	50	IOC	100%	97%	92%	100%

In our study, we are observing the diagnostic accuracy of EUS versus ERCP which is the gold standard in detecting choledocholithiasis. In multiple studies, the majority of individuals reported a mean age of 55-59 years with the genetic predilection of disease directed towards male gender [11,12], while one study conducted in the same accord reported female gender to be more affected [10]. Majority of individuals presented with upper abdominal pain, jaundice, dilated CBD stones, acute or biliary pancreatitis, ultrasound or CT scan detections suggestive of stone [11,12]. The predominant category reported among sufferers of CBD stones was the intermediate-risk category [10,11], while in a study conducted in Taiwan, males suffered more than females and the majority of patients were categorized as high-risk patients. EUS detected CBD stones in a maximum number of high-risk patients as compared to intermediate risk. The majority of patients undergoing EUS did not develop complications. The overall sensitivity, specificity, positive predictive value, negative predictive value, and accuracy were 100%, 94.7%, 91.7%, 100%, and 96.7%, respectively. CBD stone was positive in 56% (9/16) of the high-risk patients, with one false-positive case and 14.3% (2/14) of the intermediate-risk patients [14].

In a study conducted previously, the occurrence of common bile duct stones had strong gender predilection towards males when compared with females. The majority of sufferers had a mean age of 55 years (18-88 years). Maximum individuals reported abdominal pain (51%) followed by jaundice (23.4%), recent cholangitis (25.5%), and a history of acute pancreatitis (18.4%); 48.2% of patients were segregated into the high-risk category while 51.8% into the intermediate-risk category. The number of stones along with their diameters and levels of liver function enzymes were increased in the high-risk group in comparison to the intermediate-risk group. EUS detected CBD stone in 59 out of 68 patients all verified by ERCP, while EUS detected no stones in nine patients out of which seven were verified by ERCP with only two false-negative detections in individuals categorized as high risk. EUS sensitivity, specificity, positive predictive value, and negative predictive value were 96%, 100%, 100%, and 77%, respectively in the identification of typical bile duct stone in the high-risk category. In the intermediate-risk section, EUS distinguished common bile duct stone in 26 out of 73 individuals while ERCP only verified diagnosis in 24 out of 26 CBD stone positive patients giving two false positives. Conclusive sensitivity, specificity, positive predictive value, and negative predictive value of EUS in demarcating stone in the intermediate-risk category were 96%, 95.80%, 92.30%, and 97.90%, respectively. Patients undergoing EUS did not develop any post-procedure complications, in contrast to which 6.3% of individuals who underwent ERCP reported post-procedure complications, i.e., mild pancreatitis, bleeding from sphincterotomy, and sphincterotomy associated perforations thus prolonging the hospital stay [11].

In another study, males had an increased frequency of developing CBD stones than females with the majority of individuals having a mean age of 59 years. Patients included in the study with CBD stone had complained of upper abdominal pain, CT and US findings suggestive of CBD stones, bilirubin levels > or equal to 4 mg/dL, dilated CBD stones, and biliary pancreatitis. The majority of patients had a diameter of CBD stone between 0.5 cm and 1 cm. In this study patients undergoing ERCP developed complications after the procedure including minor ERCP-associated pancreatitis, elevated liver enzymes and bilirubin, septicemia, and post-sphincterotomy bleed. Patients undergoing ERCP did not develop any complications with overall sensitivity, specificity, positive predictive value and negative predictive value of the linear EUS for detecting CBD stones were 100%, 92.88%, 98.21%, and 100%, respectively [12].

One such study showed a comparison between sensitivity and specificity made on the basis of sizes and diameters of CBD stones with a maximum number of sufferers reporting with a mean size of 5.8 mm (range: 4.1-8 mm). No case was reported to be missed by EUS and detected by ERCP or vice versa. The sensitivity and specificity of EUS in detecting CBD stones > 4.0 mm and > 7.0 mm is 96% and 100%. The sensitivity of EUS in detecting stone <4.0 mm is decreased to 92% while specificity remains unchanged. In the case of diameter < or equal to 7.0 mm, both sensitivity and specificity of EUS is 100%. The specificity and sensitivity of ERCP in distinguishing stone diameter > 4.0 mm is 100% and 92%, respectively [13]. In a separate study, the mean age of patients was 47 years with female sufferers in the majority contradicting the outcome of multiple studies. The majority of sufferers belonged to the intermediate-risk category. EUS detected stone in 65.9% of patients, simultaneously these patients underwent ERCP detecting stone in all patients thus confirming the diagnosis of EUS and giving it a sensitivity of 100%. No patient undergoing EUS developed complications. In this study, EUS was found cost-effective when compared with ERCP [10].

For comparing both EUS and ERCP in terms of cost-effectiveness, EUS has increased expenses in the high-risk group and decreased expenses in the intermediate-risk group [11]. EUS was reported to be cost-effective than ERCP in all aspects by another study [10]. Patients undergoing EUS reported no post-procedure complications while those who underwent ERCP developed a variety of post-procedure complications, i.e., mild pancreatitis, bleeding from sphincterotomy, and sphincterotomy-associated perforations thus prolonging the hospital stay, an outcome supported by numerous studies [10,11,14]. A study regulated by Petrov et al demonstrates EUS lowering the risk of complications by a relative risk of 0.35, whereas avoiding ERCP in 67.1% of patients in their study with an initial EUS evaluation, thereby further reducing the risk of complication by an invasive procedure [22]. In a similar accord, many studies have persuaded the successful use of EUS and ERCP in a single session whenever both procedures are simultaneously indicated in a patient with more than a thousand cases are reported, although not yet common in routine care [23]. One study compared the use of EUS alone versus a combination of EUS and ERCP and concluded that the use of EUS before ERCP showed a significant reduction of procedure time and higher rates of successful procedures [24]. Although a well-documented editorial also highlighted the fact that initial evaluation from EUS strategy vs ERCP alone was effective in the need for eliminating an invasive procedure and was also cost-effective for 60-73% of patients suffering from choledocholithiasis [25]. Lastly, a review article concluded a sensitivity of EUS as 97%, a specificity of 98%, PPV of 100%, and NPV between 91% and 100% [26]. The limitations of the study included retrospective analysis and study participants based on a single-center experience.

## Conclusions

The gold standard for bile duct pre-operative visualization has been ERCP for decades. The non-selective application of ERCP, however, detects CBD stones with less sensitivity in all patients with suspected choledocholithiasis, also exposing them unnecessarily to an invasive procedure and a certain risk of complications. We recommend that ERCP can be selectively conducted or excluded in patients with biliary obstruction in case of EUS negative, thus minimizing the complications and morbidity associated with an invasive procedure. Recent literature has also supported the idea of an initial evaluation by EUS rather than a direct ERCP approach for patients at moderate to high risk of choledocholithiasis, which has been reported to reduce the complications associated with an invasive procedure and to be cost-effective as well by decreasing the need for an ERCP. Our results have shown comparative diagnostic accuracy of EUS in detecting biliary obstructive disease.
